# A cross-sectional study: exploring knowledge and attitude of medical and nursing students to Care for Elders in the future

**DOI:** 10.1186/s12877-022-03551-0

**Published:** 2022-11-14

**Authors:** Rawan Alqahtani, Shadan Almuhaidib, Hoda Jradi

**Affiliations:** 1 College of Public Health and Health Informatics, King Saud Bin Abdulaziz University for Health Sciences, Riyadh, Saudi Arabia; 2 Rumah General Hospital, Riyadh Second Health Cluster, Ministry of Health, Riyadh, Saudi Arabia; 3 Central Military Laboratory & Blood Bank, Prince Sultan Military Medical City, Riyadh, Saudi Arabia; 4 King Abdullah International Medical Research Center, Riyadh, Saudi Arabia; 5 Ministry of the National Guard Health Affairs, Riyadh, Saudi Arabia

**Keywords:** Knowledge, Attitude, Geriatrics, Students, Elders, Care

## Abstract

**Background:**

All societies are going through a longevity revolution. Inflating the elderly’s age group will present many challenges to the healthcare system. A better health workforce is needed to meet this demand. Little is known about the knowledge and attitude of medical and nursing students toward geriatric care in Saudi Arabia. This study aims to explore medical and nursing students’ knowledge about aging, and their attitude toward caring for older adults.

**Method:**

A cross-sectional study using two surveys: the fact on aging quiz to assess knowledge and the UCLA geriatric attitude scale to evaluate attitudes. A total of 494 medical and nursing students from three universities in Saudi Arabia were included.

**Results:**

Knowledge and attitude scores were 13.57/23 and 3.37/5, respectively. Findings showed that even in a country where the elderly are respected and family bonds are valued there is still much room for improvement. Moreover, there was a significant statistical difference in the knowledge and attitude scores (*p* < .0001) regarding the participants’ specialty. The nursing participants had higher knowledge scores, while the medical participants had better attitude scores. Overall, Spearman’s correlation coefficient between ranked knowledge and attitude scores was −.339 with a significance of *p* < .0001, indicating a low negative correlation between the two scores.

**Conclusion:**

Knowledge and attitude score were fair to moderate, these findings propose enhancing learners’ education and training experiences in the care of the elderly through curricular improvements.

**Supplementary Information:**

The online version contains supplementary material available at 10.1186/s12877-022-03551-0.

## Introduction

Aging is inevitable, in the upcoming years the aged population will inflate around the world [1, 2]. All societies are going through this longevity revolution [3, 4]. In 2019, 1 in 11 people were over the age of 65 years. This will increase to 1 in 6 people aged 65 years and more by 2050 around the world, which is about double in only 30 years.

Saudi Arabia is following the global transition regarding the expansion of the geriatric population [5]. The life expectancy at birth in the 1980s had improved from 64.4 years to 74.5 years in 2016 [5–7]. It is predicted that the Saudi older population aged 65 and above will continue to increase and will make up to 18.4% of the total population [7, 8]. This will rapidly be progressing to a demographic transition and aged population. In 2050, it is predicted that the proportion of the aged population will increase to 25% out of the population’s total number of 40 million [9]. This epidemiological transition will change patterns of mortality and morbidity in the country like elsewhere [1, 3, 10]. Changes in the population pyramid will follow and this will impose the need to raise long-term specialized care for the elderly [11].

This increasing age group will present many challenges imposed mainly on the health system which will need to uplift its services [1, 4, 12]. It will also require a better-trained health workforce to meet this demand [2, 7]. Saudi Arabia delivers free health care services to all its population [7]. It’s provided on the highest level of quality possible. However, specialized geriatric clinics along with specialized geriatricians are lacking. This is one of many challenges imposed on the older population to face. The Saudi culture is based on Islamic teachings, it ensures respect and compassion for the elderly based on the Holy Quran’s scripts. Sending them to nursing homes is viewed as abandonment and it violates the sacred duty to care for them. So as a close society the support and care must be provided by their offsprings and extended family.

Although the government still assumes that mainly the care for the elderly will be provided by family members [7], it did initiate a support system that provides financial aid, equipment, and logistical help. In addition, there are twelve social welfare homes which are like nursing homes distributed throughout the kingdom for those who don’t have a family to care for them. This service is provided by the Ministry of Human Resources and Social Development at no cost.

Another barrier to receiving healthcare is not seeking it in the first place. Mainly because changes in health are attributed to aging. Additionally, some older individuals think they will die soon so no use in seeking healthcare. Moreover, illiteracy among the elderly aged 65 years and more accounts for 59.29% [7, 13]. Therefore, elders are present at an advanced stage of illnesses, which makes health management difficult and treatment challenging [7].

Medical care for the elderly differs from that of other age groups due to age-induced changes as physiological, psychosocial related problems and often multiple comorbidities that simultaneously occur [12, 14]. Now managing comorbid conditions results in polypharmacy and iatrogenic consequences [15]. Furthermore, cancer distribution in Saudi Arabia among the elderly aged 60 years and above constituted approximately 37.5% of the total reported cancer cases in the year 2015 and this rate had increased from its previous report of 30% in 2007 [16, 17]. As the aged population increases, senility and its consequences will increase as well as the development of mild cognitive impairments and dementia. In Saudi Arabia the prevalence of cognitive impairment was estimated to be 45%, this was published in 2018 [18]. So, elders, in general, have particular care needs. They require certain knowledge about medication administration and its interactions [19]. No need to mention the level of awareness of their physiological, psychosocial, emotional, and cognitive care.

To ensure optimum care we need to support elders by providing effective services that match their needs [20, 21]. Healthcare professionals need to approach geriatrics with confidence in their knowledge and attitudes that are free of any discrimination [2]. Healthcare system reforms would take a long time to reap their benefits as well as efforts and the expenses required to re-train the healthcare workforce. Instead, focusing on medical education is a more proper and accessible way to instill gerontological principles and enhance its training thus producing a workforce who are aware of geriatric needs and are capable of accommodating them [19].

Exploring medical and nursing students’ knowledge and attitudes to care for older adults is essential for making recommendations to develop proper interventions that would match the needs of the elder community. Emphasizing attitudes in this study was supported by previous research, as knowledge alone is not enough for changing the attitudes and skills of nursing and medical students about the willingness to care for older adults [2, 22]. Attitudes are known to reflect education, culture, personal values, experience, and other characteristics of modifiable nature with adequately tailored interventions and environments [23].

The purpose of this study is to explore how prepared are medical and nursing students to care for geriatrics in the future. We aim to determine how ready they are to become geriatricians by assessing their knowledge and attitudes toward the elders.

Specific objectives:

1. To assess the knowledge about aging among medical and nursing students, using the “Facts on Ageing Quiz” by Palmore 1977, the short version [24].
2. To examine the attitudes towards the elderly among medical and nursing students, using the University of California at Los Angeles (UCLA) “Geriatric Attitude Scale” by Reuben 1998 [25].

## Materials and methods

This was a cross-sectional descriptive study design conducted among university students. The sample was obtained from medical and nursing colleges of three universities. Students are included from 1st-year till the internship period. The sampling technique was a non-probability sampling method, particularly, convenience that included all medical and nursing students who gave their consent to participate.

Data collection started at the beginning of the school year 2020-2021 year (from June till September 2020), indirectly via the college’s administrative system. The survey reached the targeted sample via e-mails, through multiple waves of communication.

Using the Raosoft sample size calculator, the recommended sample size for the current study was calculated as 323. The population size was 2000, at a 95% confidence level with a 5% margin of error, and a 50% response distribution. However, a total number of 494 students completed the questionnaire, and although the response rate was 25% the sample size exceeded the calculated minimum requirement.

The instrument used was adopted from established measurements, which were previously used in the literature for similar purposes. There were two valid and reliable tools used as outcome measures [24, 25]. First, the short version of “The Facts on Aging Quiz”; is true and false statements by Palmore [24]. The second is the “UCLA Geriatric Attitude Scale” which contains fourteen items; Likert scale (1 to 5) formatting by Reuben et al. [25]. The final version used was an electronic, anonymous, self-administered questionnaire in the English language. English is the second language in Saudi Arabia. Moreover, all medical field students have been receiving their education in English as their First language at all Saudi universities. Both tools were reviewed by expert faculties to verify their relevance to the Saudi context and their applicability and have been modified accordingly.

The final instrument was evaluated for face validity i.e., clarity, comprehension, and time management among twenty-five medical and nursing students who were not included in the studied sample. Minor adjustments were made to the wording and phrasing to simplify the language without losing core meaning. Reliability was assessed via test-retest with 90% agreement. Also, the reliability coefficient of Cronbach’s Alpha was calculated to determine internal consistency. The knowledge scale “facts on aging quiz” scored 0.64, while the “UCLA geriatric attitude scale” scored 0.60 which demonstrates an acceptable level of reliability [26, 27].

### Statistical analysis

Statistical analysis was conducted using the Statistical Package for Social Sciences program (IBM SPSS Statistics version 26), and a *p*-value of ≤0.05 was considered statistically significant.

Knowledge scale scores were based on true or false statements. A score of one indicated good knowledge and a score of zero was given for poor knowledge.

Knowledge scale answers were summed up, high score indicated good knowledge from a range of 0-23. Further, respondents’ answers to the attitudinal Likert scale (1 to 5) were summed and high scores indicated positive attitudes from a possible range of 13-65.

Descriptive statistics for continuous data were initially evaluated for normality of distribution by the Shapiro-Wilk normality test to determine the appropriate descriptive statistics either means and standard deviation or median and interquartile range (IQR). The data were not normally distributed, and the *p*-value was <.05. Thus, descriptive statistics were expressed as median, and IQR. Categorical variables were summarized and reported as frequencies and percentages.

The assessment of continuous factors associated with knowledge and attitude scores was performed using non-parametric tests: Mann-Whitney U test or Kruskal-Wallis test, as appropriate.

Univariate and multivariate linear regression analyses were applied to analyze the association between the dependent variables (knowledge and attitude of the respondents) against the social and demographic variables among medical and nursing students.

Factors found statistically significant in the univariate analysis were included in the final multivariate model. A *p*-value ≤0.05 was considered statistically significant.

Spearman’s rho correlation coefficient was used to determine the strength associations and direction between the knowledge and attitude scores.

### Scientific and ethical approval

All procedures performed in this study were following the ethical standards of the institutional research committee and with the Declaration of Helsinki and its later amendments or comparable ethical standards. The study was approved by the Institutional Review Board at King Abdulaziz University for Health Sciences located at King Abdullah International Medical Research Center (SP20/074/R). Also, IRB approval was obtained from the ethical sub-committee for humanitarian and social research at King Saud University, reference No: KSU-HE-20-351.

## Results

### Social and demographic characteristics

The total number of participants enrolled in the study was *n* = 494, of which 220 (44.5%) were males, and 274 (55.5%) were females. The participants were grouped into three age categories: 18-20 years (16%), 21-23 years (67.8%), and > 23 years (16.2%). Forty-six percent had a monthly income of < 15,000 SR, while 53.2% had a monthly income of ≥15,000 SR. The majority of the participants were from urban areas (93.3%), whereas only 6.1% lived in rural areas. Family types are predominantly nuclear in Saudi Arabia, and this was seen in our study. 73.7% of the participants lived in nuclear families, 11.5% were from single-parent families, and 10.9% belonged to extended families. The participants were selected from the medicine (*n* = 282, 57.1%) and Nursing colleges (*n* = 212, 42.9%). Sixty-seven percent (*n* = 332) did not have elderly living with them, whereas 26.1% (*n* = 129) had at least one elderly member in the family, 6.5% (*n* = 32) had two elderly members in the family, and only one participant had three elderly family members.

### Knowledge of the participants towards elderly care

The knowledge score of the participants is represented as mean ± SD in Table 1. The participants’ mean score was 13.57 ± 3.42 out of the maximum obtainable score of 23. However, the Shapiro-Wilk test (< .0001) indicated that the score was not normally distributed; therefore, the median was considered the cut-off value, which was 13.

**Table 1:** The knowledge score and responses to the knowledge questionnaire; facts on aging the short edition (*N* = 494)

**Variable**	**Mean**	**SD**	**Min**	**Max**	**Median**	**IQR**
Knowledge score	13.57	3.426	0	23	13	4
**Statements**					**N (%)**	
					**Correct answer**	**Incorrect answer**
The majority of elderly are senile					178 (36)	316 (64)
All five senses tend to decline in old age					375 (75.9)	119 (24.1)
Most elders have no interest in sex					292 (59.1)	202 (40.9)
Lung capacity tend to decline					395 (80)	99 (20)
The majority of elderly feel miserable					219 (44.3)	275 (55.7)
Physical strength tends to decline in old age					461 (93.3)	33 (6.7)
Elderly drivers have fewer car accidents					283 (57.3)	211 (42.7)
Most older workers cannot work as effectively					369 (74.7)	125 (25.3)
About 80% of the elderly are healthy enough to...					304 (61.5)	190 (38.5)
Most elders are unable to change their way of					389 (78.7)	105 (21.3)
Elders usually take a longer time to learn something					424 (85.8)	70 (14.2)
It is almost impossible for most elders to learn					148 (30)	346 (70)
The reaction time of most elderly tends to be					398 (80.6)	96 (19.4)
In general, most elderly are pretty much alike					202 (40.9)	292 (59.1)
The majority of the elderly are rarely bored					168 (34)	326 (66)
The majority of the elderly are socially isolated					196 (39.7)	298 (60.3)
Older workers have fewer occupational accidents					278 (56.3)	216 (43.7)
More than 10% of the Saudi population are now elderly					368 (74.5)	126 (25.5)
Most medical practitioner tend to give low priority					220 (44.5)	274 (55.5)
The majority of elderly have incomes below the poverty level					190 (38.5)	304 (61.5)
The majority of elderly are working or would					240 (48.6)	254 (51.4)
Elders tend to become more religious as they					444 (89.9)	50 (10.1)
The majority of elders are rarely irritated, angry					164 (33.2)	330 (66.8)

*%* Percentage, *IQR* Interquartile Range, *Max* Maximum, *Min* Minimum, *N* Frequency, *SD* Standard Deviation

Responses of the participants to the knowledge part of the questionnaire are summarized in Table 1.

### The attitude of the participants towards elderly care

The attitude score of the participants is represented as mean ± SD in Table 2. The attitude score was 43.81 ± 5.34 from the maximum possible score of 65. The scores ranged from 30 to 57. Like knowledge scores, it was not normally distributed according to the Shapiro-Wilk test (<.0001). Therefore, we considered the median, which is 43, as the cut-off value. Differences in the participants’ attitudes towards the elderly are detailed in Table 2. For simplification purposes, we have calculated the total result to be out of 5. Using this formula (43.81/ 13) and (5.34/ 13) were 3.37 ± 0.4 was the mean and the SD.

**Table 2:** The attitude score and responses to attitude statements of the UCLA geriatric attitude scale (*N* = 494)

**Variable**	**Mean**	**SD**	**Min**	**Max**	**Median**	**IQR**
**Attitude score**	43.81	5.346	30	57	43	8
**Statements**		**N (%)**				
		**Strongly disagree**	**Disagree**	**Neutral**	**Agree**	**Strongly agree**
Most old people are pleasant to be with		25 (5.1)	35 (7.1)	145 (29.4)	195 (39.5)	94 (19)
I would rather see younger patients than older ones		50 (10.1)	128 (25.9)	187 (37.9)	99 (20)	30 (6.1)
It is the family responsibility to provide care for the elderly		22 (4.5)	25 (5.1)	75 (15.2)	159 (32.2)	213 (43.1)
Medical care for elders uses up too many material and human resources		42 (8.5)	76 (15.4)	184 (37.2)	154 (31.2)	38 (7.7)
As people grow older, they become less organized and more confused		35 (7.1)	142 (28.7)	147 (29.8)	142 (28.7)	28 (5.7)
Elderly patients tend to be more appreciative of the medical care I provide than are younger patients		9 (1.8)	39 (7.9)	192 (38.9)	170 (34.4)	84 (17)
Taking a medical history from elderly patients is frequently troublesome		18 (3.6)	65 (13.2)	186 (37.7)	184 (37.2)	41 (8.3)
I tend to pay more attention and have more sympathy towards the elderly patients than the younger patients		22 (4.5)	55 (11.1)	170 (34.4)	178 (36)	69 (14)
Elders, in general, do not contribute much to society (as in work, social events, and family gathering)		85 (17.2)	141 (28.5)	139 (28.1)	113 (22.9)	16 (3.2)
Treatment of chronically ill old patients is hopeless		149 (30.2)	165 (33.4)	105 (21.3)	69 (14)	6 (1.2)
Elders do not contribute towards paying for their health care		60 (12.1)	154 (31.2)	176 (35.6)	91 (18.4)	13 (2.6)
In general, old people act too slow for modern society		37 (7.5)	69 (14)	173 (35)	190 (38.5)	25 (5.1)
It is interesting when listening to the elder’s past experiences		15 (3)	18 (3.6)	66 (13.4)	134 (27.1)	261 (52.8)

*%* Percentage, *IQR* Interquartile Range, *Max* Maximum, *Min* Minimum, *N* Frequency, *SD* Standard Deviation

### Sociodemographical factors influencing respondents’ knowledge and attitude regarding elderly care

Table 3 shows the relationship between the socio-demographic characteristics to the mean ranks of knowledge and attitude scores. For instance, the 18–20-year age group had the highest mean rank (278.33) in knowledge with a significance of <.0001, whereas no significant difference was seen in attitude mean rank in terms of age. Average monthly income showed a significant influence on both knowledges (*p* < .0001) and attitude (p < .0001). However, an inverse relationship was seen between income and knowledge, whereas a positive relationship was seen between income and attitude. The participant’s origin and family type did not play any significant role in the knowledge or attitude scores.

**Table 3:** Comparison of social and demographic characteristics, and median and mean ranks of knowledge and attitude scores (*N* = 494)

Variable	N	%	Knowledge score				Attitude score			
			Median	IQR	Mean rank	P	Median	IQR	Mean rank	P
Gender										
Male	220	44.5	13	11-15	213.62	**<.0001**	44.5	41-48	273.93	**<.0001**
Female	274	55.5	14	12-16	274.71		42	40-46	226.28	
Age (years old)										
18-20	79	16	15	12-16	278.33	**<.0001**	43	39-46	237.54	.519
21-23	335	67.8	13	12-16	255.47		43	40-47	246.24	
> 23	80	16.2	12	10.25-14	183.66		44.5	40-48.75	262.60	
Monthly income										
**<** SR 15,000	231	46.8	14	12-16	277.52	**<.0001**	42	40-46	222.12	**<.0001**
**≥** SR 15,000	263	53.2	13	11-15	221.13		44	41-48	269.79	
Origin										
Urban	464	93.9	13	12-16	247.81	.847	43	40-48	246.67	.609
Rural	30	6.1	13	12-15	242.65		43	42-47	260.38	
Family type										
Nuclear	364	73.7	13	11-15	241.77	.053	44	40-48	253.68	.238
Extended	54	10.9	14	11-16.25	270.57		42	40-45	216.69	
Grand parents	4	0.8	15	12.75-18	323.13		42.5	35.75-44	180.00	
Single parents	57	11.5	13	12-15.5	233.91		43	40-49	253.11	
Others	15	3	15	14-18	334.97		41	38-47	205.20	
Specialty										
Medicine	282	57.1	13	11-15	209.45	**<.0001**	44	41-49	276.21	**<.0001**
Nursing	212	42.9	15	12-17.75	298.11		42	39-45	209.31	
Specialty x Gender										
Medicine x Male	187	37.9	13	11-14	200.80	**<.0001**	45	41-48	282.58	**<.0001**
Medicine x Female	95	19.2	13	12-15	226.48		44	41-49	263.67	
Nursing x Male	33	6.7	14	12.5-17	286.24		43	38-47	224.92	
Nursing x Female	179	36.2	15	12-18	300.30		42	39-45	206.43	
Year										
1st	2	0.4	16	.	343.50	**<.0001**	38.5	.	92.00	.256
2nd	3	0.6	15	.	342.00		45	.	281.17	
3rd	140	28.3	15	12.25-16	292.59		43	40-47	244.62	
4th	140	28.3	13	12-16	252.14		43	40-46	232.55	
5th	72	14.6	13	11-15	236.85		43	40-46	247.23	
6th	49	9.9	13	11.5-	231.51		47	40-51	283.29	
Internship	88	17.8	12	15.5	180.60		44	40-48	258.55	
				10-14						
Family number										
1-3	34	6.9	14	13-16	285.72	.207	42	40-47	225.96	**.018**
4-6	229	46.4	13	11-16	239.94		44	41-48	267.06	
**≥** 7	231	46.8	13	12-16	249.37		43	40-46	231.28	
Elders number										
0	332	67.2	13	12-16	244.92	.524	43	40-48	248.93	.096
1	129	26.1	13	11.5-16	255.60		44	40.5-48	258.10	
2	32	6.5	13	11-15	236.20		41.5	39-44.75	194.81	
**≥** 3	1	0.2	Omitted	Omitted	422.00		Omitted	Omitted	90.00	

*%* Percentages, *IQR* Interquartile Range (25th, 75th), *N* Frequency, *p*-value

A significant difference was seen in the knowledge and attitude scores (*p* < .0001) regarding the participants’ specialty. The nursing participants had a higher mean rank in the knowledge scores, while the medical participants had a better mean rank in the attitude score. The scores varied significantly as combined stratification was applied between gender and specialty. Female nurses had the highest knowledge score, and in general, females had better knowledge scores compared to males in the same specialty. While males who specialized in medicine had the highest attitude score. In general, males had better attitude scores compared to females in the same specialty.

The scores further varied significantly (*p* < .0001) with the year groups in college, seen in relation to the knowledge mean rank. However, no significant relation was seen between attitude and the year groups. It was further observed that the first year had the highest mean rank, and the interns had the lowest. Moreover, participants with large families had the highest mean rank in the attitude scores, with significant variation observed with the family type (*p* < .018), but no significance was seen in terms of knowledge. Lastly, the number of elders living in the same household did not significantly influence the knowledge or attitude scores.

Beta coefficient (β) was reported in the univariate and multivariate linear regression analysis model and presented statistical significance for some factors for both the knowledge and attitude scores.

Details on the regression model for factors associated with the knowledge score can be seen in Table 4. The age factor in the > 23 years group had a statistically significant inverse relationship in both the univariate (*p* < .0001) and multivariate model (*p* ≤ 025), respectively. Moreover, nursing and medicine specialties had a statistically significant inverse relationship (*p* < .0001) with knowledge in both univariate and multivariate models. Origin, family type, the year that the participants were in, the number of family members living in the same household, and the number of elders did not present any significant statistical value.

**Table 4:** Univariate and multivariate linear regression analysis model for variables associated with knowledge score (*N* = 494)

Variable	Knowledge score				
	N (%)	Univariate analysis β (95% CI for B)	P	Multivariate analysis β (95% CI for B)	p
Gender					
Male	220 (44.5)	−.21 (−2.02 to −.83)	**<.0001**	−.020 (−.84 to .57)	.71
Female	274 (55.5)	Reference		Reference	
Age (years old)					
18-20	79 (16)	Reference		Reference	
21-23	335 (67.8)	−.07 (−1.33 to .33)	.237	−.003 (−.85 to .81)	.96
> 23	80 (16.2)	−.24 (−3.24 to − 1.14)	**<.0001**	−.132 (−2.30 to −.16)	**.025**
Monthly income					
**<** SR 15,000	231 (46.8)	Reference		Reference	
**≥** SR 15,000	263 (53.2)	−.189 (−1.90 to −.70)	**<.0001**	−.068 (− 1.10 to .166)	.148
Origin					
Urban	464 (93.9)	Reference		–	
Rural	30 (6.1)	.007 (−1.17 to 1.37)	.877		
Family type					
Nuclear	364 (73.7)	Reference		Reference	
Extended	54 (10.9)	.047 (−.46 to 1.50)	.297	−.02 (− 1.18 to .74)	.649
Grand parents	4 (0.8)	.046 (−1.62 to 5.12)	.307	.03 (−2.29 to 4.58)	.514
Single parents	57 (11.5)	−.052 (−1.51 to .40)	.255	−.084 (−1.82 to .03)	.058
Others	15 (3)	.109 (.40 to 3.93)	**.016**	.074 (−.21 to 3.16)	.087
Specialty					
Medicine	282 (57.1)	−.315 (−2.76 to −1.60)	**<.0001**	−.288 (−3.06 to −.927)	**<.0001**
Nursing	212 (42.9)	Reference		Reference	
Year					
1st	2 (0.4)	Reference		–	
2nd	3 (0.6)	−.008 (−6.32 to 5.65)	.913		
3rd	140 (28.3)	−.197 (−6.16 to 3.18)	.530		
4th	140 (28.3)	−.297 (−6.93 to 2.41)	.343		
5th	72 (14.6)	−.26 (−7.24 to 2.16)	.288		
6th	49 (9.9)	−.26 (−7.65 to 1.81)	.226		
Internship	88 (17.8)	−.44 (−8.63 to .744)	.099		
Family number					
1-3	34 (6.9)	Reference		–	
4-6	229 (46.4)	−.152 (−2.28 to .19)	.098		
**≥** 7	231 (46.8)	−.129 (−2.12 to .35)	.16		
Elders number					
0	332 (67.2)	Reference		–	
1	129 (26.1)	.037 (−.41 to .99)	.412		
2	32 (6.5)	−.002 (−1.27 to 1.23)	.972		
**≥** 3	1 (0.2)	.046 (−3.24 to 10.26)	.308		

95% CI 95% Confidence interval, *β* beta coefficient, *p*-value

Details on the regression model for factors associated with the attitude score can be seen in Table 5. The participants’ specialty upholds its significant positive relationship in both univariate (*p* < .0001) and multivariate (*p* < .001) models for attitude. All other factors either presented significance in the univariate model or manifested no significance in the univariate nor multivariate analysis.

**Table 5:** Univariate and multivariate linear regression analysis model for variables associated with attitude score (*N* = 494)

Variable	Attitude score				
	N (%)	Univariate analysis β (95% CI for B)	P	Multivariate analysis β (95% CI for B)	p
Gender					
Male	220 (44.5)	.15 (.68 to 2.56)	**.001**	.02 (−.94 to 1.31)	.745
Female	274 (55.5)	Reference		Reference	
Age (years old)					
18-20	79 (16)	Reference		–	
21-23	335 (67.8)	.041 (−.85 to 1.79)	.486		
> 23	80 (16.2)	.059 (−.81 to 2.53)	.313		
Monthly income					
< SR 15,000	231 (46.8)	Reference		Reference	
≥ SR 15,000	263 (53.2)	.16 (.77 to 2.64)	**<.0001**	.09 (−.06 to 2.04)	.065
Origin					
Urban	464 (93.9)	Reference		–	
Rural	30 (6.1)	.004 (−1.89 to 2.08)	.924		
Family type					
Nuclear	364 (73.7)	Reference		–	
Extended	54 (10.9)	−.078 (−2.86 to .200)	.088		.
Grand parents	4 (0.8)	−.055 (−8.53 to 2.01)	.224		
Single parents	57 (11.5)	.012 (−1.30 to 1.69)	.796		
Others	15 (3)	−.058 (−4.58 to .948)	.198		
Specialty					
Medicine	282 (57.1)	.209 (1.32 to 3.19)	**<.0001**	.26 (1.12 to 4.53)	**.001**
Nursing	212 (42.9)	Reference		Reference	
Year					
1st	2 (0.4)	Reference		–	
2nd	3 (0.6)	.104 (−2.39 to 16.73)	.141		
3rd	140 (28.3)	.44 (−2.29 to 12.62)	.174		
4th	140 (28.3)	.40 (−2.76 to 12.16)	.216		
5th	72 (14.6)	.36 (−2.05 to 12.97)	.154		
6th	49 (9.9)	.39 (−.63 to 14.48)	.072		
Internship	88 (17.8)	.40 (−1.94 to 13.03)	.146		
Family number					
1-3	34 (6.9)	Reference		–	
4-6	229 (46.4)	.14 (−.48 to 3.37)	.14		
≥ 7	231 (46.8)	.02 (−1.69 to 2.16)	.81		
Elders number					
0	332 (67.2)	Reference		Reference	
1	129 (26.1)	.02 (−.88 to 1.30)	.706	.07 (−.32 to 1.91)	.164
2	32 (6.5)	−.09 (−3.89 to −.014)	**.048**	−.06 (−3.27 to .56)	.166
≥ 3	1 (0.2)	−0.4 (−15.39 to 5.61)	.36	−.06 (− 16.94 to 3.51)	.198

95% CI 95% Confidence interval, *β* Beta Coefficient, *p*-value

Spearman’s rho correlation coefficient was calculated to measure the strength and direction of the association, if any, between ranked knowledge and attitude scores. The value of r_s_ is −.339, with a significance of *p* < .0001, indicating a low negative correlation between the two scores.

## Discussion

This study explores the knowledge and attitude of medical and nursing students toward caring for older adults. Knowledge scores of study participants were (mean = 13.57, median = 13) which appears to be higher when compared to another regional study where nurses scored 11.13 as their average [28]. However, when we compare it with western studies, our results are not that satisfactory. For example, a study on baccalaureate nursing students resulted in a mean score of 16.5. According to Palmore, on average undergraduate students scores 14, and nurses in practice score 16.5. Similarly, other studies have also reported that the mean scores in the borderline range from 17.3 to 18.3 [29–31]. This indicates that the overall knowledge score of our study participants was low meaning geriatric education was insufficient [11].

It was observed in our study that knowledge correlates significantly with age and specialty. Where the younger the participant the more knowledgeable he/she appears to be. This agrees in concept but differs in the result with Lambrinou et. Al. who reported in their study that students in their final year had better knowledge about older clients so the age of the participants influences knowledge positively [32].

To our knowledge, no studies compared medical and nursing students, the nursing students were more knowledgeable than medical students. However, medical students had better attitudes toward older adults where they scored more than average 3.37/5 but in comparison to Fitzgerald et al. they had a better score than ours 3.7/5 where the higher the score the better the result and hence the positive the attitude [22]. Moreover, this agrees with Lee et al. but several studies have reported otherwise, indicating that nursing students’ attitudes were more positive towards older people [21, 33, 34]. In addition, none of the other characteristics was significate like experience [25]. On the contrary Fitzgerald et al. reported that caring for the elderly before medical school is associated with more positive attitudes toward them [22]. In addition, several other surveys have demonstrated that senior health care students have more positive attitudes towards older clients than younger students [32, 35]. At the start of their education, the negative attitude of students could be due to their limited knowledge and experience with the elderly. The negative attitude should improve with increased experience, exposure, and professional knowledge involving the elderly.

Religion and culture play an important role in shaping healthcare services [4]. In Saudi Arabia, the family social norms are influenced by religion, old age is often observed to be respected and honored [8]. Islam as a religious system affects how the elderly are perceived and encourages Muslims to respect and value the elderly. Elders also prefer the involvement of their families in caring for them. So, prospective individuals are raised to be caring towards their elders. Despite the socio-economic and cultural changes in Saudi Arabia due to modernization and urbanization, respect for the elderly is still a prominent feature in society.

A slight inverse relationship was seen in the knowledge-attitude correlation. In theory, clinicians with higher education and greater knowledge about geriatrics and gerontology should present better attitudes. They are better at anticipating and identifying the elderly’s needs than those without any professional knowledge in this area. Nevertheless, an inverse correlation between the level of education of the students and their attitudes was observed. A study in Poland reported that it is common for subjects with a higher level of education or those taking part in the extramural program to present a negative attitude. It seems possible that in terms of extramural students, having long-term professional experience, their negative attitude would stem from the difficult working conditions, work overload of professional duties, or simply job burnout [36]. However, a study by Hweidi and Al-Hassan carried out in Jordan did not support the correlation between knowledge and attitude reported in the Polish study [37]. They reported that the participating nurses in the study demonstrated a moderately positive attitude toward older patients in acute care settings, and years of clinical experience correlated significantly with their positive attitudes [35]. According to a study, the quality of elderly care is directly associated with the care providers’ attitudes. Negative attitudes toward the elderly can affect the quality of care adversely, so it is crucial that nursing and medical students learn to approach the care of the elderly with a positive attitude and view their health and aging as being natural [35, 38–41].

A more detailed assessment of the medical field students’ experiences is needed to differentiate and measure their level of competency in addition to studying attitudes and knowledge patterns among various health specialties that have close contact with geriatrics [21, 22, 42]. In addition, nursing and medical curricula must include intensive gerontological/geriatric education and training to promote knowledge of anticipating elders’ needs and enhance attitudes towards elderly care in preparation for the increment of the older population [10, 19, 20, 23, 32, 35, 37, 43, 44]. There should be a focus on positively influencing the learners’ experience before and during the medical school education period through meaningful participation in caring for older adults [12, 20, 23, 41]. As well as future reinforcement to maintain the progress of quality care [45, 46]. Lastly, governmental, and institutional initiatives and policies on a broad scale are needed to establish specialized geriatric clinics with renounced geriatricians that provide holistic care and allow exposure periods to targeted groups. Also, establishing contact with the younger ages such as middle and high school could positively influence individuals’ and community attitudes toward the elderly and aging [47, 48]. Furthermore, exploring the prevalence of ageism and discrimination against the elders in the healthcare system and assessing the elders’ satisfaction with the healthcare services is another aspect to be uncovered [49].

### Strength and limitation

A major strength of this study that it enriches the Saudi Arabian context where it is lacking and evaluate the need for improvement more locally within the community. This will help improve the healthcare services provided to the geriatric population since they are a growing group.

This study had some limitations. Mainly, the generalizability of this research finding is limited due to the non-probability sampling technique that was used.

## Conclusions

Knowledge and attitude scores were fair to moderate in comparison to other studies. Further, a positive attitude towards the elderly wasn’t prevalent among the study sample. Overall, there was a slight inverse relationship between knowledge and attitude. These findings advocate for enhancing students’ curricula and training in gerontology. Moreover, increasing the number of geriatricians and geriatric clinics for enriching the learners’ experiences in the care of the elderly.

Further studies are needed to explore the views of the elderly on healthcare services to enrich available literature in Saudi Arabia. Identifying the elders’ and the students’ views will help the healthcare system in adapting to future demands.

## Supplementary Information

**Additional file 1.**

**Additional file 2.**

## Data Availability

The datasets used and/or analyzed during the current study are available from the corresponding author upon reasonable request.
