# Supplementary material for: A cross-sectional study: exploring knowledge and attitude of medical and nursing students to Care for Elders in the future
Source: BMC Geriatr. 2022 Nov 14;22:856. doi: 10.1186/s12877-022-03551-0 (PMC9664597; doi:10.1186/s12877-022-03551-0)
Supplement: Supplementary file 1 — Additional file 1. [file 12877_2022_3551_MOESM1_ESM.docx]

| **Demographic characteristics** | | |
| --- | --- | --- |
| Sex:  ☐ Male ☐ Female | | |
| Age:  ☐ 18-20 ☐ 21-23 ☐ More than 23 | | |
| Family type:  ☐ Nuclear family  ☐ Single family  ☐ Extended family  ☐ Grandparents family  ☐ Other; …………… | Class:  ☐ 1^st^ year  ☐ 2^nd^ year  ☐ 3^rd^ year  ☐ 4^th^ year  ☐ 5^th^ year  ☐ 6^th^ year  ☐ Internship | Specialty:  ☐ Medicine  ☐ Nursing |
| Number of family members:  ☐ 1-3  ☐ 4-6  ☐ 7+ | Place of origin:  ☐ Urban  ☐ Rural | Affiliation:  ☐ KSAU-HS  ☐ KSU  ☐ Qassim Uni.  ☐ Others;……….. |
| Average level of income:  ☐ Less than 5,000  ☐ 5,000-9,999  ☐ 10,000-14,999  ☐ 15,000-19,999  ☐ 20,000-24,999  ☐ More than 25,000 | Number of elders at home:  ☐ 0  ☐ 1  ☐ 2 | |

| **T** | **F** | **Facts on Aging Quiz** |
| --- | --- | --- |
|  |  | 1. The majority of old people (past age 65) are senile (i.e. defective memory, disoriented, or demented).  2. All five senses tend to decline in old age.  3. Most old people have no interest in, or capacity for, sexual relations.  4. Lung capacity tends to decline in old age.  5. The majority of old people feel miserable most of the time.  6. Physical strength tends to decline in old age.  7. Aged drivers have fewer accidents per person than drivers under age 65.  8. Most older workers cannot work as effectively as younger workers.  9. About 80% of the aged are healthy enough to carry out their normal activities.  10. Most old people are set in their ways and unable to change.  11. Old people usually take longer to learn something new.  12. It is almost impossible for most old people to learn new things.  13. The reaction time of most old people tends to be slower than reaction time of younger people.  14. In general, most old people are pretty much alike.  15. The majority of old people are seldom bored.  16. The majority of old people are socially isolated and lonely.  17. Older workers have fewer accidents than younger workers.  18. Over 10% of Saudi population are now age 65 or over.  19. Most medical practitioners tend to give low priority to the aged.  20. The majority of older people have incomes below the poverty level.  21. The majority of old people are working or would like to have some kind of work to do (including housework and volunteer work).  22. Older people tend to become more religious as they age.  23. The majority of old people are seldom, irritated or angry. |

| **Geriatric Attitude Scale Statements** | **Strongly**  **Disagree** | **Somewhat**  **Disagree** | **Neutral** | **Somewhat**  **Agree** | **Strongly**  **Agree** |
| --- | --- | --- | --- | --- | --- |
| 1. Most old people are pleasant to be with. | 1 | 2 | 3 | 4 | 5 |
| 2. I would rather see younger patients than elderly ones. | 1 | 2 | 3 | 4 | 5 |
| 3. It is society’s responsibility to provide care for the elderly. | 1 | 2 | 3 | 4 | 5 |
| 4. Medical care for old people uses up too much human and material resources. | 1 | 2 | 3 | 4 | 5 |
| 5. As people grow older, they become less organized and more confused. | 1 | 2 | 3 | 4 | 5 |
| 6. Elderly patients tend to be more appreciative of the medical care I provide than are younger patients. | 1 | 2 | 3 | 4 | 5 |
| 7. Taking a medical history from elderly patients is frequently an ordeal. | 1 | 2 | 3 | 4 | 5 |
| 8. I tend to pay more attention and have more sympathy towards my elderly patients than my younger patients. | 1 | 2 | 3 | 4 | 5 |
| 9. Old people in general do not contribute much to society. | 1 | 2 | 3 | 4 | 5 |
| 10. Treatment of chronically ill old patients is hopeless. | 1 | 2 | 3 | 4 | 5 |
| 11. Old persons don’t contribute their fair share towards paying for their health care. | 1 | 2 | 3 | 4 | 5 |
| 12. In general, old people act too slow for modern society. | 1 | 2 | 3 | 4 | 5 |
| 13. It is interesting listening to old people’s accounts of their past experiences. | 1 | 2 | 3 | 4 | 5 |
